# Exploring work-life balance among professional women in mainland China: A qualitative study

**DOI:** 10.3389/fpsyg.2022.938664

**Published:** 2022-11-09

**Authors:** Ying Pan, Gong Sun

**Affiliations:** ^1^International Business School Suzhou, Xi'an Jiaotong-Liverpool University, Suzhou, China; ^2^Management School, University of Liverpool, Liverpool, United Kingdom; ^3^School of Business, Changshu Institute of Technology, Changshu, China

**Keywords:** professional women, work-life balance, barriers, resources, qualitative research, mainland China

## Abstract

This article introduces a qualitative study utilizing semi-structured interviews to explore the barriers and resources of professional women to attain work-life balance (WLB) in the context of mainland China. Interviews were conducted with full-time employed women who had received higher education and possessed professional qualifications (*N* = 20). The findings reveal underlying factors in work and non-work domains which influence the achievement of work-life balance among Chinese professional women. Participants were found to perceive and attain their work-life balance differently according to their personal traits. The findings also highlight social and family support resources for Chinese professional women from the perspective of Confucianism. This study contributes to current knowledge on work and life issues through the lens of professional women's work-life balance perceptions and experiences in mainland China.

## Introduction

After the People's Republic of China (PRC) was founded in 1949, Chairman Mao and the central government launched a campaign to encourage women to pursue higher education and take on more significant positions at work (Li, 2021; Zhu et al., 2022). A Chinese saying states that “women hold up half the sky” which is related to recent history in China (Li, 2021; Zhang et al., 2021). With the increasing education levels of women in China, the number and proportion of professional women are growing (Zhang et al., 2021). Chinese women are employed in an increasing number of different fields and occupations (Zhu et al., 2022).

The rapid transformation of industrialization and globalization has had a significant impact on many aspects of employment and worker welfare. However, due to the influence of globalization, women's role as housekeeper has not been weakened, which makes professional women have the dual roles of professional women and housekeeper (Zhang et al., 2020). Based on the statistics of the Guangming Online (2022) the average weekly working hours of employees in enterprises are 48.0 h nationwide. According to statistics from the National Bureau of Statistics of China (2019a), the average time for Chinese residents to accompany and care for their children was 3 h and 8 min, including 2 h and 17 min for men and 3 h and 31 min for women. These factors are associated with welfare indicators such as organizational turnover and physical and mental health burdens (Wang and Li, 2017; Li et al., 2018). Consequently, throughout the past 20 years, women's experiences in organizations have grown to be a prominent area of study (Opara et al., 2020).

Conducting WLB research in the context of China offers a unique opportunity. First, as a traditional collectivist society, China values the importance of community and family. It focuses on fulfilling social roles and responsibilities to the family in groups. Second, women play a major role in China as a global power. In 2018, female employees accounted for 43.7% of the national workforce (National Bureau of Statistics of China, 2019b). There are 742 female deputies in the 13th National People's Congress, accounting for 24.9% of the total number (National Bureau of Statistics of China, 2019b). Moreover, the proportion of women on corporate boards of directors accounted for 39.9% in 2018, an increase of 7.2% compared with 2010 (National Bureau of Statistics of China, 2019b). Such a high proportion of working women has brought about changes in the organization and family structures and roles of women. Third, for more than 5,000 years, the philosophy of Confucianism has played a dominant role in expected Chinese behavior and standards (Le et al., 2020). Chinese women are still expected to handle major domestic work for families, even though they work full-time (Chen et al., 2018; Zhang et al., 2021). Therefore, professional women have a dual burden of both work and family.

This study aims to advance our understanding of work-life issues among Chinese professional women. Despite the increasing empirical studies on work-life research in the West, there is limited attention focused on the Asian labor force in this research field, thus this study must take a closer look at work and life in Asia (Le et al., 2020). This study seeks to explore Chinese professional women's work-life balance from an indigenous perspective and clarify women's active participation in the labor market, household division, and care provision in the context of the modern Chinese family. Based on semi-structured interviews with 20 professional women in China, this research contributes to the debate and evidence from previous studies by providing insights into how Chinese professional women experience and relate to the intersection between work and life. Because the specific experiences of Chinese professional women and their intersectional experiences within the Chinese context are not always consistent with the experiences of women in western countries, this study focuses on specific claims about Chinese professional women and their intersectional experiences within the Chinese context.

## Literature review

Work-life balance (WLB) is a central concern in daily work and life discourse (Greenhaus et al., 2012). The definition of “work” is often straightforward while the concept of “life” is more diffuse. Life in this research refers to private life outside of work (Hagqvist et al., 2020). In this study, WLB was conceptualized as an individual's perceptions of how well his or her life roles are balanced (Greenhaus et al., 2012). From this point of view, individuals measure the balance between work and the rest of their life subjectively (Haar et al., 2014) which is contrary to the dominant opinions that consider balance to be objectively measured through a low level of role conflict, high role enrichment, or equal distribution of time and attention among several roles that constitute an individual's life (Frone, 2003). The definition of WLB is based on a perception-centered approach which holds that WLB is a general concept unique to everyone, depending on their life values, priorities, and goals (Kossek et al., 2012; Haar et al., 2014).

Most previous studies have focused on work-family balance, regardless of the individuals' broader lives including community, leisure, church, sports, and other activities (Hall et al., 2013). The majority of the research evidence in the field of work-life balance shows that the conflict between work and life is exacerbated by long working hours, high job demands, high workload, and high work responsibility which professional women often face (Hagqvist et al., 2020).

China is a unique country because the philosophy of Confucianism has had a substantial impact on the cultural values of China (Le et al., 2020). However, a closer look at the experiences of women in China suggests that gender equality has not been realized (Zhang et al., 2020). Although gender role expectations in China are changing, gender role traditions endure (Zhang et al., 2021). The previous studies outline the significant role of traditional Chinese culture in shaping women's work values with some positive effects on the career advancement of women that may coexist with negative effects (Zhang et al., 2021).

A profession is an occupation that satisfies the prerequisites of extensive education and training, formal testing of ability, restricted entrance, professional associations, a code of conduct, and a sense of responsibility to serve the public (Roberts, 2005; Opara et al., 2020). The professional women in this research refer to the full-time female employees with an undergraduate degree or above, who undertakes job and possess technology or management skills and create value for their enterprises (Opara et al., 2020; Zhang et al., 2020).

This study draws on boundary theory which discusses the way people construct, maintain, negotiate, and cross the boundaries between work and family roles (Ashforth et al., 2000). Boundary theory refers to the individual creation and maintenance of “boundaries as a means of simplifying and orderly the environment” (Ashforth et al., 2000). Boundary theory is mainly used in the research fields of human resource management and organizational behavior. It is often used to explore the impact of work-life conflict on workforces and how to avoid or weaken this impact through WLB. According to the boundary theory, individuals can create and maintain segmented or integrated boundaries between work and family fields actively (Xie et al., 2022). As the needs and purposes of work and family are often different, the comprehensive boundary usually leads to negative consequences for employees, including low family satisfaction and serious psychological pain, such as emotional exhaustion (Li et al., 2018; Xie et al., 2022).

Although there is research evidence regarding the imbalance of work and family responsibilities among Chinese women,little is known about how Chinese professional women understand and experience the interface between work and life. Thus, how women feel about their lives is important. Since WLB has been proven to be a prominent topic among Chinese professional women, this research adopted an inductive approach to explore Chinese women's WLB. Aligned with the situationist perspective, this research poses two research questions:

**Research Question 1**: What are the barriers affecting how Chinese professional women to attain WLB?
**Research Question 2:** What are the resources Chinese professional women relying on to maintain WLB?

## Methods

Scholars have called for increasing the use of qualitative methods to study work-life balance issues (Phillips et al., 2016; Adisa et al., 2019; Dutta, 2020; Hwang and Beauregard, 2022). An interpretivist approach helps us understand the feelings and experiences of women (Kvale and Brinkmann, 2015). We employed an exploratory qualitative research approach due to the emphasis on how individuals interpret their social world (Bryman, 2016). By employing a combination of purposive and convenience sampling, semi-structured interviews were conducted to collect data so that we could retain an open mind about the notions and theories that might arise from the data (Bryman, 2016).

### Participants

Participants were sourced through various channels such as emails, social media groups (WeChat), and alumni associations. Four criteria had to be met simultaneously for an individual to be considered as a participant. First, each woman should have obtained undergraduate education or above. Second, the participant should possess occupational qualifications, which is a necessary characteristic for professional women. Third, women should have full-time jobs in the workforce and be financially independent. Last, they should all work and live in mainland China. The average age of the participants was 39 years and their ages ranged from 24 to 50 years. The interviewees worked in various industries which include manufacturing, agriculture, public management, finance, transport, and so on. In total, 10 of the participants held bachelor's degrees, nine of them had master's degrees, and one had a doctor's degree. The participants came from a wide range of Chinese cities, being from the Yangtze River Delta, and residing in cities such as Shanghai, Nanjing, and Suzhou. The demographic characteristics of the participants are shown in Table 1.

**Table 1:** Demographic characteristics of the participants.

**ID**	**Age**	**Work experience**	**Marital status**	**Number of children**	**Occupation**	**Yes/No work-life balance**	**Working hours per day**	**Time for parenting per day**
A1	37	10	Divorced	1	Finance Analyst	Yes	8 h	6 h
A2	38	15	Married	1	Personnel Manager	Yes	8 h	8 h
A3	37	13	Married	2	Deputy Director of Office	Yes	7 h	5 h
A4	37	13	Married	1	Product Manager	No	12 h	4 h
A5	38	14	Single	0	Director of Corporate Culture Department	Yes	8 h	0 h
A6	42	18	Married	1	Sales Director	Yes	8 h	6 h
A7	43	12	Married	1	Entrepreneurs	No	10 h	4 h
A8	40	13	Married	2	Entrepreneurs	No	9 h	3 h
A9	42	18	Divorced	0	Accountant	Yes	8 h	0 h
A10	29	6	Married	1	Department Manager	No	9 h	5 h
A11	40	12	Married	2	Engineer	No	9 h	4.5 h
A12	35	8	Married	1	Doctor	No	9 h	2.5 h
A13	39	16	Married	1	Product Manager	No	8 h	5 h
A14	50	27	Married	1	Associate Professor	No	6 h	4 h
A15	45	23	Married	2	Accountant	No	7 h	7 h
A16	41	13	Married	1	Lecturer	No	9 h	5 h
A17	48	23	Married	2	Department Vice President	No	8 h	3 h
A18	37	12	Married	0	Sales Manager	Yes	8 h	0 h
A19	36	12	Married	1	Brand Founder	Yes	9 h	2 h
A20	27	3	Single	0	Project Leader	No	9 h	0 h

### Procedures

The authors conducted one pilot interview to become familiarized with the interview schedule. Each participant received an introductory email from us to invite them to take part in the study. A consent form and an information page were also attached to the invitation. Participants were made aware of the confidentiality and anonymity of the interviews. No personal data such as a name, phone number, or other details were collected or registered. Informed consent was obtained from all the participants and respondents were provided with the opportunity to understand the nature of the research and the implications of their participation from the beginning (Bryman, 2016).

We conducted semi-structured interviews with 20 professional women. The interviews were conducted face-to-face or *via* remote audio chat provided by WeChat individually (Tan et al., 2021). WeChat is one of the most popular social media platforms in China (Montag et al., 2018). We selected audio chat because of the belief that the interviewees could feel more comfortable when they discuss their personal feelings and stories in an anonymous manner (Tan et al., 2021). The interviews were performed from October 2020 to April 2021. Five participants were interviewed face-to-face and 15 by WeChat interview. All the interviews are synchronous. Using online interviews to ask sensitive questions is more effectively compared to offline interviews because interviewees may feel less anxious to answer questions when they are not physically present (Bryman, 2016). Through face-to-face interviews, we can elicit questions, and ask *in situ*, that can be closely related to the participants' everyday experiences (Sand et al., 2022). In this study, the interviewees could choose the way they wanted to conduct the interviews. Considering the impact of COVID-19, 5 women participated in on-site interviews and 15 women chose WeChat voice chats. Interviewers asked the scripted questions initially, with verbatim phrasing used to maintain consistency. Then, interviewers asked follow-up questions to clarify responses and investigate issues in greater depth (Phillips et al., 2016). All the conversations were conducted in Mandarin, with a few involving regional dialects or a mixture of Chinese and English. These interviews were recorded electronically and lasted around 40–60 min on average. The data collection was ended until no new insights were found and evidence became repetitive and we determined that data saturation had been reached (Strauss and Corbin, 1998).

### Data analysis

The interviews were transcribed verbatim, including all possible speech errors. NVivo 12 was used to conduct data analysis. The authors read the interview text several times to be familiar with the content, then coded line by line according to the research questions (Fereday and Muir-Cochrane, 2006). This research follows the approach developed by Strauss and Corbin (1990) to represent and classify coding into open coding, axial coding, and selective coding.

#### Open coding

The data were broken down into a large number of codes, we undertook naming and conceptualization as part of the open coding process (Zhang et al., 2021). There were 23 topics connected to barriers to attaining work-life balance and resources to maintain work-life balance were discovered.

#### Axial coding

We analyzed the results of the open coding process in the axial coding process, categorizing the 23 concepts into nine categories: multiple roles, family pressure, work-centric organizational culture, Chinese traditional stereotype, personal traits, family support, social support, family-supportive organizational culture, and government support.

#### Selective coding

**Figure 1:**
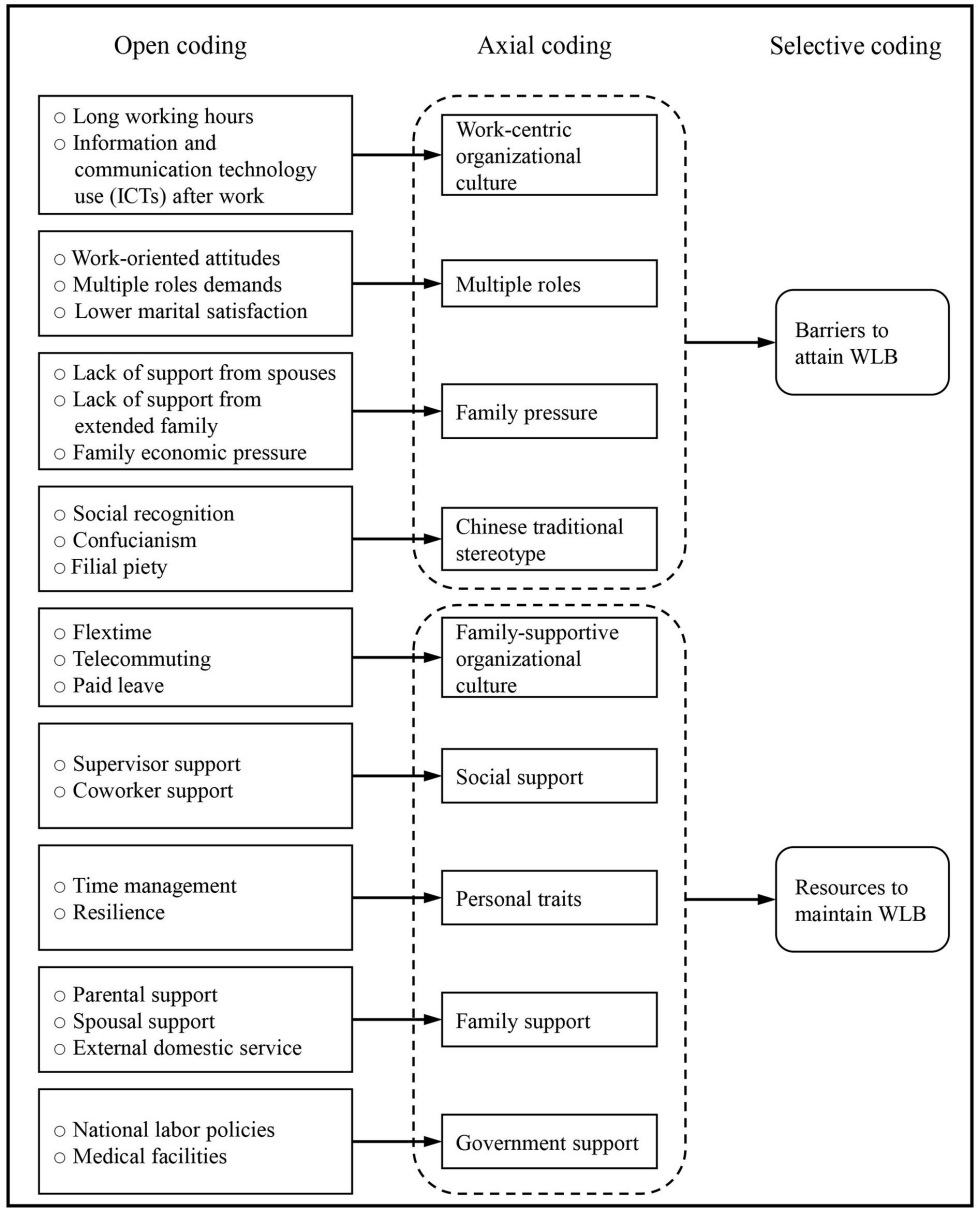
Data analysis structure.

Selective coding is the process of sorting through categories to find the ones that are most relevant to research. We chose two of the nine categories at this point which are barriers to attaining work-life balance and resources to maintain work-life balance (Figure 1).

## Results

Various barriers to attaining work-life balance were identified from the findings. Multiple roles, family pressure, work-centric organizational culture, and Chinese traditional stereotype were revealed as four types of barriers for Chinese professional women to attain work-life balance.

According to the study's findings, 12 Chinese professional women reported they were not in a status of work-life balance. Their work activities frequently occur in the non-work domain, whereas non-work activities do not frequently occur in the work domain, which contrasts with findings in Western countries (Phillips et al., 2016; Kossek et al., 2021). Overtime work is highly prevalent and serious in China. The longer the working hours, the less care they can take for their families (Zhao and Zhang, 2019). The conflict between work and family in China is also a fierce collision of values (Pan et al., 2022). China advocated a collectivism culture in which hard work, self-sacrifice, and contribution to society were regarded as the highest embodiment of individual value (Lin et al., 2021). Although the female employment rate in China is much higher than the world average, the pattern of division of labor within the family has not changed significantly. The traditional pattern of division of labor in the family is still popular in China, and women continue to be the primary caregivers in their households (Cooke, 2022). In other words, Chinese professional women share the responsibility with their spouses economically. In terms of housework, women pay much more than men. The work-family conflict faced by Chinese women is even more serious.

Work-family conflict is viewed as a conflict between roles by Western scholars, who emphasize the subjective experience of individuals involved in the conflict (Greenhaus and Beutell, 1985), whereas work-family conflict in China is viewed as a fundamental conflict between income needs and personal life responsibilities (Yuan et al., 2022).

Professional women in China desire a higher standard of living at this time in their development, but they are also under a lot of financial strain, such as house loan, medical care, and education, resulting in a widespread sense of economic strain among the participants (Cooke, 2022). With more and more Chinese dual employees entering the workforce, earning extra money through overtime work has become an unavoidable option for many families (Chen et al., 2018; Huang et al., 2019). If professional women reduce their working hours or their spouses leave the workforce, the financial strain on families will worsen. Chinese professional women's work-life balance may encounter increased barriers due to the aging population and postponed retirement policy in China.

## Barriers to attain work-life balance

Two core categories of work and non-work domain barriers became apparent. Some of these barriers were the same as those uncovered by previous research in Western countries, whereas others appeared unique to Chinese professional women, such as work-centric organizational culture, and Chinese traditional stereotypes.

### Work domain barriers

The results showed that the main barrier for professional women to attain work-life balance is the work-centric organizational culture. Some organizations ask employees to work remotely with electronic equipment which creates a high-pressure working atmosphere within the company and seriously affects women's work-life balance.

#### Work-centric organizational culture

The dynamics of the workplace are shifting, and work-family conflict is further aggravated as a result of developments such as the shift to a 24/7 economy and the increasing influence of technology (Heras et al., 2020; Wang et al., 2020). Organizational culture prizes work overtime and prioritize work over family, creating barriers to achieving work-life balance. Due to the rapid changes in work environments caused by the rise of managerialism and economic globalization, work-life conflicts for finance, health care, and social service employees have increased significantly.

According to the results of the interviews, 15 respondents reported work-centric organizational culture as a barrier. The average working hours were 8.45 h per day, while according to the People's Republic of China Labor Law (2019), the State shall practice a working hour system wherein laborers shall work for no more than 8 h a day which means the participants work quite long hours. Long working hours and information and communication technology used after work extend work duties into the non-work domain, which blurs work and life boundaries. In the work and life domains of these Chinese professional women, low levels of job control led to increased stress-related mental health issues and decreased work-life balance (Wang and Li, 2017). About 12 women reported that they frequently work extra hours and they complained about technology use after work hours blurring the line between their work and private life.

As A4 shared, “I have stressful jobs and long working hours; sometimes I think my work is endless. Actually, I'm always working at night around 11 p.m. I don't have enough time to spend with my daughter. She's already asleep when I arrive home.”

For example, A6 recounted:

I was in one well-known company 6 years ago. I worked overtime nearly every working day and usually stayed up late to 1 or 2 am to deal with the work. As you know, smartphones and laptops hamper the peace of life after work. I often received calls from my leaders or colleagues to discuss work at night or on weekends. My leisure time is limited, and I don't have the time to spend with my husband and my son.

Most of the interviewees were subject to the 996 working hour system which is a work schedule in China some companies practice that requires workers to work from 9 a.m. to 9 p.m., 6 days per week, for a 72-h work week, and they complained about uncertain working hours and expectations of mandatory unpaid overtime. A5 explained the excessive working demands she encountered:

Our company's requirement is 24-hour standby for accidents. Sometimes, although I am off work, there are some urgent accidents that need to be dealt with quickly, so I have to go to the scene of the accident. These accidents are unpredictable, you may not be familiar with all the locations in such a large geographical area, so it will take you a lot of time to drive there. You don't know when the work will be finished.

The views expressed by professional women reinforced a Confucian ideal of distance and power. They reported male staff members being preferred over female ones due to perceived higher levels of ambition and work ethic. They also admitted that women, especially those who are married and with children, are more likely to go home earlier. However, the volume of work often forced these women to work late, not leaving their offices in the evenings before their leaders, because their leaders expect them to place work before their personal lives. For example, A20 described her challenge with competition in the workplace:

The competition in my industry is too fierce. Some female colleagues even switch to working online after taking maternity leave. If you want to succeed in the workplace, you have to work harder than your male colleagues and be more excellent than them. As the competition is relatively high, there is little time for me to consider my own private life.

### Non-work domain barriers

Some barriers concerning multiple roles, family pressure, and Chinese traditional stereotypes were identified. Many participants admitted that they have difficulties and obstacles in achieving WLB due to the multiple roles they juggle.

#### Multiple roles

Especially for the interviewees with children, the most challenging factor was their children's dependency on them. Totally 13 out of 16 working mothers reported this as the most common challenge they faced in their life domain. According to A7, the demands of motherhood immediately after giving birth are increased. As A12 stated, “My daughter constantly attaches herself to me and hopes that she can spend more time with me, while the heavy workloads prevent me from spending enough time with my daughter.” Meanwhile, A13 stated that she often takes on the responsibility such as washing her daughter's clothes and taking medicine when she is sick.

Most professional women with work-oriented attitudes are less focused on satisfying their needs. The development of their careers also creates a challenge to maintaining WLB. Women who are more successful in their profession are less likely to have satisfying lives. In addition, many women abandon what they perceive to be “superfluous” desires, such as early bedtimes and leisure time. According to A8, who is a founder of educational institutions, “It was very hard at the beginning of starting a business. I had to think about everything like costs and recruitment. I hardly have time to watch movies with my children on weekends.”

#### Family pressure

The study also noted that marriage may encourage men to specialize in market work while women may be more likely to take on more child-rearing responsibilities. 16 interviewees have one or two children and they have to take care of their children outside of work. According to statistics from the National Bureau of Statistics of China (2019a), the average time for Chinese residents to accompany and care for their children was 3 h and 8 min, including 2 h and 17 min for men and 3 h and 31 min for women. The average time of the interviewees for parenting is 3.7 h which increased their burden. A13 stated, “I often work overtime in my company. When I get home, my husband and my daughter are asleep. My daughter's homework is often left on the table and waiting for me to check.” She then said that she was angry because her husband did not help her with her daughter's homework. Although personal circumstances affect women's WLB to different extents, the effects on their mental health are significant and unmistakable.

Spouses' work demands can affect the WLB of Chinese professional women due to the husbands' frequent business travel, long working hours, and hours that do not overlap. Many of the married interviewees noted that their spouses were busy with their own work and could not share the increasing demands at home which generally made it hard for them to achieve WLB. A3 shared her experience, “My husband works in Beijing and often comes home once a month or on holidays. I live in our hometown with our two sons. Most of the time, I can only solve problems at home by myself.”

A14 said:

My husband has been working in a city out of my living area and only comes back on weekends. Sometimes he has meetings and activities on weekends and can't come back. He often receives an urgent call even though he's supposed to spend time with us during the weekends. I usually ask my sister and brother-in-law to help me if I need help.

#### Chinese traditional stereotype

The results of the study revealed that traditional social recognition still has a large impact on the attitudes toward women's domestic responsibilities. If a woman has high achievement motivation, she will be criticized for not being able to take care of her family and housework. People around her will also criticize her for not being able to raise a family. For example, A7 said, “Chinese traditional social recognition supports the idea that women should stay at home after getting married. It also states that a woman should be able to spend enough time with her children to make them happy.”

## Resources to maintain WLB

### Work domain resources

#### Family-supportive organizational culture

The study revealed that family-supportive organizational culture can help professional women perform better in organizations. It can also help women develop their personal relationships and build a stronger WLB. In some companies, flexible scheduling can also help women work more effectively. According to A18, she has benefited greatly from the flexible working schedule of her company. It allows her to work from home and take advantage of the company's technical equipment.

Family-supportive organizations also provide women with psychological support and help them through difficult times. A1 highlighted the organizational culture in her company and stated, “My company provides ultimate support in terms of career growth, freedom and the flexibility. I enjoy my job because it allows me to pursue my career and family at the same time.”

#### Social support

Most participants agreed that social support and relationships at the workplace are very important factors that help improve WLB. For instance, 14 out of 20 participants identified supervisor support as a noteworthy resource that helped them achieve better WLB. As A13 elaborated:

I benefit a lot from the family-supportive organizational culture. If my daughter has a cold or some accidents at school during work hours, I will communicate with my department leader who understands my situation well. I can take my annual leave to take care of my child.

Meanwhile, A15 indicated that being recognized by her managers helped her take on new projects and reduced her company's expenses.

### Non-work domain resources

#### Personal traits

The most mentioned determinants shaping the WLB of professional women include time management and resilience.

The ability to manage time is also a key factor that professional women consider when it comes to achieving a better WLB. This is evident in the following quote of A19: “I usually work with my daughter when she has classes on weekends. She has two classes in the morning or in the afternoon, so I can make use of that time to complete my own work with my laptop.” Similarly, A16 stated that “I usually do my work when my kids are at school, nursery or after they go to bed, or I would get up early in the morning before my children wake up. Now I and my kids get up together when we can, they keep their sleep schedule and I do my job. It works quite well.”

During the process of participating in the workplace, Chinese professional women have gradually formed their own resilience and subject consciousness by contributing to society, allowing them to have greater self-control over their work and life. A11 noted, “I personally feel that men and women are equal. My husband has his work and I have mine. If I can control the work environment, I can do better in my job.” A15 elaborated that she would sacrifice her non-work time to take care of her children, rather than seek help from parents, “I want to educate my son within the ideas of our generation. Although it's harder, it will also reduce the burden for my parents.” This was confirmed by A6 as well, who said:

Now I switched to the position of sales. I think the barriers of work-life conflict have been solved very well, because I have a lot of spare time. I can pick my son up from school in the afternoon. I can talk with him about what difficulties he encountered in school during dinner, and I can solve the problems in time.

#### Family support

Family support was also a major factor that contributed to WLB in Chinese professional women. Family support fills the lack of non-market labor aid caused by women's entry into the workplace. Several issues concerning family support resources were identified, such as parental support, spousal support, and external domestic service.

The majority of women received parental support which could lighten the burden of their life. The results revealed that 16 professional women achieved WLB by relying on parental support from their elder parents who live with them. And 15 of them live with their parents and 1 lives with her parent-in-law and mother-in-law. Only one married woman did not have children, and two married women with children live in a nuclear family. One divorced woman with children lived with a hired helper. Elder parents often help women by sharing the home duties such as childcare and house chores, which can reduce the domestic housework burden of professional women and allow more time for both work and leisure activities. Extended households with three generations with a spouse, at least one of their parents, and their unmarried children were a common living arrangement for the majority of the participants, especially those with children. These results reveal that professional women in mainland China more often turn to their elderly parents rather than relying on hiring helpers for outsourcing domestic work.

For instance, A19 said that her parents and parents-in-law help her with the housework and childcare duties by picking her children up from school and cooking dinner in the evening. They also help with daily cleaning. She stated that her parents' help greatly reduces her burden of housework.

Parents also support their daughters' career development. As A3 elaborated, “My mother supports me working in my favorite career and encourages me to pursue my passion. Without her encouragement, I could not achieve a balanced life or work well.”

Spouses can provide instrumental and emotional support to individuals, which can help minimize the negative effects of work overload. Many women also stated that their partners provide them with the support they need in the workplace and in life. For instance, A10 said that her husband often gives her encouragement when she gets into trouble at work:

My husband gives me a lot of encouragement. If I encounter trouble, he will support me immediately. He always encourages my achievements in work. If I am stressed by work, he will console me and discuss how to solve the problem which will make me more comfortable at home and enhance my work-life balance.

A6 also confirmed that “My husband has given me a lot of support to achieve personal growth in my career.”

#### Government support

The legitimate rights of women are protected by various laws and regulations which include the Labor Law of the People's Republic of China and the Marriage Law of the People's Republic of China. Chinese women enjoy equal rights with men in political, economic, cultural, social, and family life, such as health protection, occupational training, social insurance and welfare, and other labor security.

As A17 elaborated, “The Labour Law has helped women get more support from the government. It also encourages the development of spiritual civilisation by protecting the rights of women to work and live a balanced life.”

## Discussion

The findings of this research contribute to the theoretical void by exploring the WLB of full-time professional women in a Chinese context. As contextualization enables researchers to understand culturally embedded constructs (Chuang et al., 2015), this context is a particularly fruitful advantage for obtaining a new theoretical understanding of WLB. The results show that the major barriers to WLB for Chinese professional women include excessive working demands and work-centric organizational culture. An improved work environment protects employees' job satisfaction and psychological wellbeing (Cooper et al., 2019). Another finding is that Chinese professional women perceive WLB in terms of resource allocation, including time and attention in work and non-work fields and a sense of achievement both at and outside work, which may mean something different for people in different personal life stages with different personal values.

The current study uncovered the substantial role of parents in mitigating the burden of housework and raising children, which in turn supports professional women while they engage in the labor market. The explanation for this phenomenon is that it is common for married couples to live with the parents of one spouse in China (Le et al., 2020). This shift might be due to the one-child policy in China which changed the family pattern from the traditional big family to the present nuclear family, which increases the attachment of parents and their child. These results are in accord with previous studies indicating that employees in collectivist cultures rely on help from extended family members to achieve domestic duties (Stock et al., 2016). However, such cohabitation modes may increase conflicts such as disagreements about childcare which may still affect professional women's WLB satisfaction.

Work-life balance in China has some characteristics that differ from those in Western countries and cannot be explained merely by the related Western work-life balance theory. In general, the work-life balance of Chinese professional women is inextricably linked to the present economic development stage in China and the distinct Chinese cultural characteristics. The western work-family conflict theory contains clear endogeneity characteristics of the family such as the life cycle changes (Greenhaus and Beutell, 1985). In contrast, the current barriers for Chinese professional women to attain work-life balance are characterized by an external, structural issue, and the majority of barriers experienced by employees are tied to some structural source. The trend of labor-intensive industries exacerbates Chinese professional women's work-life conflict. China has adopted a labor-intensive growth approach for many years (Liu and Zhang, 2022). Countries that implement labor-intensive growth strategies typically artificially decrease employees' income and reduce workers' welfare. The current national income distribution pattern in China is characteristic of this scenario, the salary costs decreased in the government and firms under pressure. Furthermore, the expansion of college enrollment, combined with the development of technology, innovation, and service industries in China resulted in an annual increase in the number of college graduates which lead to the supply of talent outnumber the demand for talent in the labor market, and overall employment competition is too severe.

Moreover, the work-life balance of Chinese professional women is tightly linked to Chinese traditional culture, both in terms of attaining it and overcoming its barriers. In modern China, the opposing principles of “work first” and “family first” are held simultaneously (Kinglun et al., 2014). China promoted a collectivism culture in which hard effort, self-sacrifice, and contribution to society were viewed as the highest embodiments of individual value (Le et al., 2020). Since the reform and opening up in 1978, this value of dedication and sacrifice, combined with capital investment, has resulted in slogans such as “take the enterprise as your home,” “proud of the factory,” and other slogans containing the concept of time deprivation being widely promoted in organizations, and the entire society has a high tolerance for harsh labor conditions such as overtime work (Zhang et al., 2020). Second, Chinese traditional culture places a strong emphasis on the importance of family (Zhang et al., 2021). The findings show that family happiness and filial piety are essential emblems of Chinese professional women's personal integrity and that three generations living under one roof is widespread in China (Qi, 2021).

### Theoretical implications

This study contributes to the current literature by informing our understanding of work-life research in mainland China. Our findings show that family support and social support have strong influence in China. These results corroborate the findings of a great deal of the previous work on cultural factors of the work-life interface.

Confucianism maintains a significant influence on the cultural values of professional women in China. The philosophy of Confucius also influenced the social positions and role perceptions of individuals in society (Van Norden, 2003). Cultural values and norms play a role in shaping the work-life interface. According to the philosophy of Confucius, the husband is responsible for the financial demands of the family, while the wife is responsible for caregiving and housework (Le et al., 2020), but these stereotypes hamper the career development of Chinese professional women. Hard work, resilience, and loyalty are respected in the philosophy of Confucianism (Zhang et al., 2020). Thus, professional women with work-oriented attitudes tend to spend long hours at work to complete work demands, although this may interrupt their private lives and ability to get support from family members (Le et al., 2020). Professional women need to have harmonious relationships with their family members, colleagues, and supervisors (Li et al., 2018).

The findings of this study also support recent studies indicating that collectivistic societies encourage individuals to rely on their family members for support (Le et al., 2020; Zhang et al., 2020). Social support can enhance individuals' self-esteem which in turn helps them obtain better career achievement and protect them from emotional exhaustion, which indicates that fostering supportive environments should help enhance the quality of life (Li et al., 2018). Notably, the relationship between employees and their employers is also viewed as a family relationship (Chen et al., 2018), which explains how the participants reduced their work-family conflict through support from their families and supervisors. Future studies on the cultural values and perspectives of Chinese professionals could use these factors as moderators or control variables to analyze the effects of their actions on work-life experiences.

### Practical implications

The findings of this research provide valuable insights to enhance Chinese professional women's WLB. The 996 work system is still common in China. As women continue to enter the labor market, they should seek further support under the background of demographic changes and marketization (Cooke, 2022). The study revealed that working after regular office hours is a major problem for professional women in China. It also revealed that the lack of support from their organizations can affect their WLB. The findings of this study, therefore, suggest that a supportive culture is needed to help professional women maintain healthy WLB. A supportive leader's behavior can help build a better relationship between subordinates and encourage them to treat one another with dignity and respect. In light of this fact, managers need to cultivate positive working relationships with employees, recognize the development potential of subordinates, and care for subordinates' lives (Zhao et al., 2019). Employees will be encouraged to make additional efforts to reciprocate supervisors' support and trust (Zhao et al., 2019). Supervisors should understand the various factors that influence the development of a supportive work environment to improve the effectiveness of organizations' training programs.

Intergenerational care can better alleviate the work-family conflict faced by women as intergenerational care can not only improve the labor supply of women with young children (Du et al., 2018; Kang and Liu, 2022) but also improve the female fertility rate (García-Morán and Kuehn, 2017; Zhao and Zhang, 2019). Especially in China, there is a serious contradiction between the supply and demand of formal childcare services (Yang et al., 2022). Therefore, intergenerational care is a more feasible and realistic choice for most professional women. First, the supply of formal childcare services is seriously inadequate (Hu and Yuan, 2022). For example, the supply of childcare services for 0- to 3-year-old children is almost blank and the number of public kindergartens for 3- to 6-year-old children is relatively rare (Hu and Yuan, 2022; Yang et al., 2022). Second, the childcare services lack flexibility in the time arrangement. Most of the childcare institutions close early and are not synchronized with their parents' working hours, which is difficult for parents to take into account (Kang and Liu, 2022). Third, childcare institutions in China are mostly daycare and are closed on both weekends and public holidays, working mothers still have to spend a lot of time and energy in the evening and weekend care, which does not effectively reduce their childcare burden (Li et al., 2020).

There are also individual differences when it comes to WLB barriers or resources and how they affect the boundaries of work and life. In terms of resources, most professional women benefit from family support which reduces their burden in their life domain. Accessing high levels of support from within the family can improve women's performance in their workplace and allow them to achieve more career success. Therefore, developing strategies as a family is important for enhancing professional women's WLB.

### Limitations and future research directions

The potential limitations of the study provide opportunities for future research. First, due to the scope of social contact and the limited classification of contact groups, the extent of the generalization of the findings of this research is restricted. Therefore, future research may enlarge samples in broader regions with a cross-sectional design. Second, from the perspective of multiple definitions of WLB, work demands and time allocation issues generate specific barriers to women's energy allocation which rely on the active interventions of the organization. Related to the findings, building a good work environment, maintaining a positive workplace, and having family support can help women balance their work and life better. Therefore, how the organizations and family members help women and alleviate WLB barriers should be further explicated with empirical studies. Third, some interviewees noted that their personal attributes can affect their WLB. Due to the increasing number of scholars who believe that WLB has a significant impact on an individual's wellbeing, future research must also look into how these attributes influence and affect WLB. To gain a deeper, comprehensive understanding of the WLB of working women, it will also be necessary to adopt quantitative research or mixed method designs.

## Conclusion

This study explored the barriers and resources Chinese professional women encounter in their attempts to maintain WLB in mainland China. The findings indicate that work and life issues are a prominent concern for Chinese professional women. Chinese professional women's WLB is influenced by a complex interaction of work domain barriers and non-work domain barriers. Long working hours and excessive work demands have been found as consistent predictors of poor WLB (Liu et al., 2021). During this qualitative study, it became apparent that women have their wisdom and resources they use to navigate their daily work and life responsibilities and maintain their relationships, thus proving that their personal traits also help them confront barriers to professional achievement and life goals. Thoughts on the influence of organizational culture and family assistance on WLB have been raised for possible directions in future research.

## Data availability statement

The raw data supporting the conclusions of this article will be made available by the authors, without undue reservation.

## Ethics statement

The study involving human participants was reviewed and approved by University Ethics Committee (UEC) of Xi'an Jiaotong-Liverpool University. The participants provided their informed consent to participate in this study.

## Author contributions

YP conceived the idea, developed the theoretical framework and worked on the literature review, contributed to the design of the study, collected data, analyzed data, and wrote and edited the manuscript. GS assisted in the research and revised the manuscript. Both authors contributed to the article and approved the submitted version.

## Conflict of interest

The authors declare that the research was conducted in the absence of any commercial or financial relationships that could be construed as a potential conflict of interest.

## Publisher's note

All claims expressed in this article are solely those of the authors and do not necessarily represent those of their affiliated organizations, or those of the publisher, the editors and the reviewers. Any product that may be evaluated in this article, or claim that may be made by its manufacturer, is not guaranteed or endorsed by the publisher.
